# Manual of Commercial Methods in Clinical Microbiology

**DOI:** 10.3201/eid0901.020623

**Published:** 2003-01

**Authors:** William J. Martin

**Affiliations:** Tucson, Arizona

During the past 25–30 years, tremendous strides have been made in the development of various commercial methods designed to simplify the isolation (in some cases) and the detection or identification (in most cases) of many different microbes in the laboratory. During these years, the time-honored conventional test methods have served the overall science of microbiology well. However, in a clinical microbiology laboratory, speed and accuracy are essential because the specimen must be processed and the results returned to the requesting physician by yesterday, if possible. Thus, many of the commercial methods reviewed in this book were developed mainly for use in the clinical microbiology laboratory, providing both rapid and accurate results with a minimum of hands-on use.

To my knowledge, this reference manual is the first resource that covers all subdisciplines of clinical microbiology. The book contains 18 chapters, including separate chapters on molecular microbiology, emerging infectious diseases, information management, and veterinary clinical microbiology, as well as chapters on bacterial identification and antimicrobial susceptibility testing, blood cultures, mycology and mycobacteriology, virology, and parasitology. A chapter on licensure and regulation of commercial products is also included, which I found helpful. In addition, the book provides an appendix that lists the manufacturers and distributors for many of the systems described in the book. The authors include a description of the sensitivities, specificities, and predictive values of the tests from peer-reviewed sources. Another chapter of interest focuses on future technology for the clinical microbiology laboratory. My only suggestion is that future editions of this text include a chapter on the history of how all these commercial tests came into being, instead various authors alluding to this point in their respective chapters.

Each chapter is well referenced, and many chapters contain tabular material that is, for the most part, easy to read and understand. The photography is adequate, although several photographs are blurred and lack clear definition. Although the intended audience for this book is primarily clinical microbiologists and other professionals who work in these environments, I suspect that many physicians, including infectious disease specialists, will find this book especially valuable when deciding what tests to order for their patients, especially in light of the high costs of health care.

As with any multi-authored text, some unevenness in the writing is expected. However, I believe that the overall scope and format of this book are quite useful, and that readers will find this manual a valuable, comprehensive source of information. The authors are to be commended for tackling such an enormous project and successfully presenting it in such a readable format.

